# Clinical significance of microRNAs in chronic and acute human leukemia

**DOI:** 10.1186/s12943-016-0518-2

**Published:** 2016-05-14

**Authors:** Chien-Hung Yeh, Ramona Moles, Christophe Nicot

**Affiliations:** Department of Pathology, Center for Viral Oncology, University of Kansas Medical Center, 3901 Rainbow Boulevard, Kansas City, KS 66160 USA

## Abstract

Small non-coding microRNAs (miRNAs) are epigenetic regulators that target specific cellular mRNA to modulate gene expression patterns and cellular signaling pathways. miRNAs are involved in a wide range of biological processes and are frequently deregulated in human cancers. Numerous miRNAs promote tumorigenesis and cancer progression by enhancing tumor growth, angiogenesis, invasion and immune evasion, while others have tumor suppressive effects (Hayes, et al., Trends Mol Med 20(8): 460–9, 2014; Stahlhut and Slack, Genome Med 5 (12): 111, 2013). The expression profile of cancer miRNAs can be used to predict patient prognosis and clinical response to treatment (Bouchie, Nat Biotechnol 31(7): 577, 2013). The majority of miRNAs are intracellular localized, however circulating miRNAs have been detected in various body fluids and represent new biomarkers of solid and hematologic cancers (Fabris and Calin, Mol Oncol 10(3):503–8, 2016; Allegra, et al., Int J Oncol 41(6): 1897–912, 2012). This review describes the clinical relevance of miRNAs, lncRNAs and snoRNAs in the diagnosis, prognosis and treatment response in patients with chronic lymphocytic leukemia (CLL), chronic myeloid leukemia (CML), acute lymphocytic leukemia (ALL), acute myeloid leukemia (AML) and acute adult T-cell leukemia (ATL).

## Background

### Chronic lymphocytic leukemia (CLL)

CLL is characterized by slow growth and the accumulation of incompetent CD5+, CD19+ and CD23+ B lymphocytes in blood, marrow, and other lymphoid tissues. This disease can be distinguished into aggressive and indolent subtypes with deletion of chromosome 13q14 being the most common genetic alteration found at diagnosis.

#### miRNA signature in CLL

The miR-15/16 cluster, miR-34b/c, miR-29, miR-181b, miR-17/92, miR-150, and miR-155 represent the most frequently deregulated miRNAs reported in CLL, and these microRNAs have been associated with disease progression, prognosis, and drug resistance [1] (Table 1). Nearly two-thirds of CLL cases presented a down-regulation of miR-15a/16-1 expression. In fact, miR-15a and miR-16-1 are located in the locus 13q14.3, a genomic region frequently deleted in CLL patient samples [2]. However, additional mechanisms, such as overexpression of histone deacetylases (HDACs), also down-regulateed expression of miR-15 and miR-16 [3]. An inverse correlation between miR-15a/16-1 and BCL2 expression has been reported in CLL, and inhibition of this microRNA expression in leukemic cell lines led to increased BCL2 expression and resistance to apoptotic signals. Comparative microarray analysis in CLL patients with high or low levels of miR-15a/16-1 identified a gene signature that contains the anti-apoptotic BCL2 family member MCL-1, which was associated with B-CLL cell survival and chemotherapy resistance [4–6]. Down-regulated miR-15a and miR-16-1 in CLL patients has been associated with a good prognosis, consistent with previous reports that correlated 13q14.3 deletions with a favorable course of CLL [7].

**Table 1:**
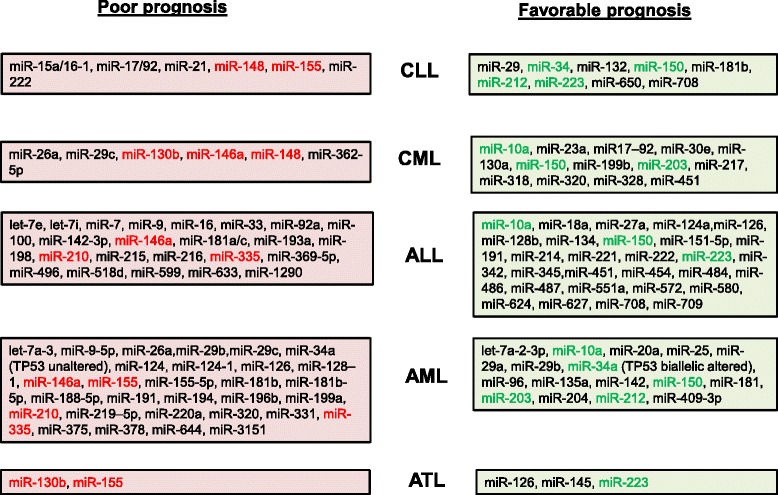
microRNAs deregulated and associated with clinical outcome in human Leukemia

The figure represents a summary of miRNAs associated with a poor or a favorable prognosis in CLL, CML, ALL, AML and ATL. Highlighted in red and green are the miRNAs that are found most frequently associated with an unfavorable or favorable outcome, respectively, across different human leukemias

The miR-29 family, which includes miR-29a, miR-29b and miR-29c, was also significantly down-regulated in a subset of CLL patients and was associated with an unfavorable prognosis. miR-29b targets DNA methyltransferase (DNMT) isoforms and inhibition of miR-29b expression may lead to hypermethylation and epigenetic silencing of several tumor suppressors [7] (Table 2). In addition, evidence showed that miR-29 targets the oncogene T-cell leukemia 1 gene, TCL1A, which was overexpressed in patients with unmutated immunoglobulin heavy chain variable regions (IgVH) and involved in translocations and inversions characteristic of mature T-cell prolymphocytic leukemia (PLL). Transgenic mice that overexpressed TCL1 in B cells displayed a similar phenotype to aggressive forms of human CLL [7].

**Table 2:**
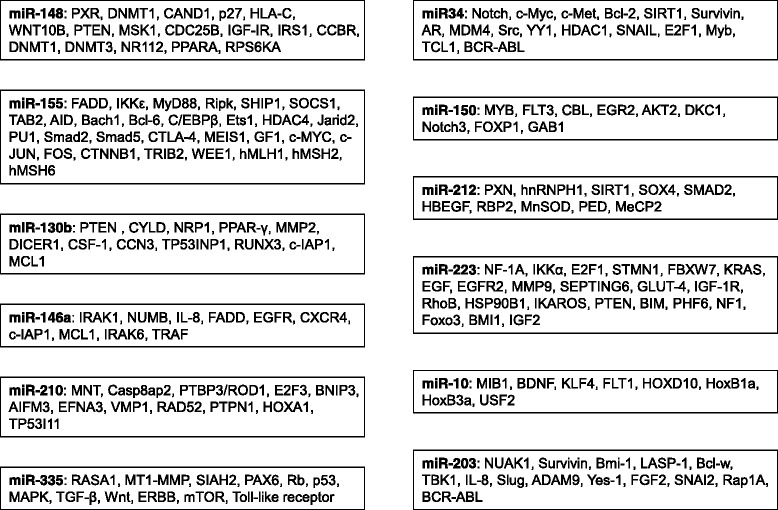
microRNAs deregulated in human leukemia and their predicted target genes

microRNAs most frequently deregulated in human leukemia (CLL, CML, ALL, AML and ATL) and their characterized target genes

Another genomic region frequently deleted in CLL patients was found in the 11q region where a miR-34 cluster is located. In fact, down-regulation of miR-34a in CLL has been associated with p53 inactivation, impaired DNA damage response, and apoptosis resistance [8–10]. Since miR-34a also inhibited E2F1 and B-Myb [11], loss of miR-34a expression may increase tumor cell proliferation. In contrast, the miR-17/92 polycistronic microRNA cluster was overexpressed in several lymphoid malignancies and inhibits the expression of the pro-apoptotic factor Bim and the tumor suppressor PTEN [12]. Activation of STAT3-induced IL-6 in tumor cells stimulates the expression of miR-17 and miR-19a, resulting in lower expression of TLR7 and TNFα.

In addition, CLL patient cells expressing zeta-chain-associated protein 70 kDa (ZAP-70) have demonstrated significantly lower levels of miR-150 expression when compared with ZAP-70-negative CLL cells. In CLL cells miR-150 targeted forkhead box P1 (FOXP1) and GRB2-associated binding protein 1 (GAB1), thereby reducing B-cell receptor signaling [13].

Another STAT3-activated microRNA, miR-155 [14], has been overexpressed in cells and in circulating microvesicles in CLL samples [14]. Induction of the onco-miR-155 in the plasma of CLL patients correlates with poor response to treatment and disease progression and, consistent with this, patients who achieved complete remission presented low levels of miR-155 in the plasma [1]. In addition, the expression of miR-155 was increased with disease progression from monoclonal B-cell lymphocytosis (MBL) to CLL and was higher in MBL and CLL than normal controls [15]. Given this, high expression of miR-155 was associated with a poor clinical prognosis in CLL [16, 17].

Finally with regard to miRNAs and CLL, miR-181b was frequently down-regulated in CLL patients with disease progression [18, 19] as it targets MCL-1 and BCL2 [18], which are important for cancer cell survival, and low expression of this miRNA was associated with poor prognosis as indicated by treatment-free survival (TFS) [18, 19]. Interestingly, a recent study based on whole genome sequencing of CLL patients identified 8 somatic mutations of miR-142 in five cases, although the role of these mutants in CLL pathogenesis is unclear [20].

#### miRNA expression and drug response in CLL patients

Higher expression of miR-650 and miR-708 has been associated with a favorable CLL prognosis [21] and affects B-cell proliferation [22]. This is in part explained by the fact that artificial miR-650 (MIMIC miR-650) reduced CLL cell proliferation through targeting cyclin-dependent kinase 1 (CDK1), inhibitor of growth 4 (ING4), and early B-cell factor 3 (EBF3) [22]. In addition, ectopic expression of miR-708 suppressed the NF-κB signaling pathway through targeting IKKβ and reduced the phosphorylation of IκBα and expression of NF-κB target genes [21]. On the other hand, overexpression of miR-21, miR-148a, miR-155 and miR-222 in CLL patients was associated with poor therapeutic response and prognosis [15–17, 23, 24]. For example, the expression of miR-155 was higher in CLL patients that failed to achieve a complete response to a chemo-immunotherapy combination of fludarabine [24, 25], cyclophosphamide, and rituximab (FCR) [15], and it was associated with poor clinical prognosis in CLL [16, 17]. Relapsed patients have higher miR-155 expression compared to baseline despite reduced expression at the beginning with response. Ectopic expression of miR-155 increases the response to B-cell receptor (BCR) ligation, which may explain the oncogenic role of miR-155 in CLL. Analyzing the gene expression profile reveals that miR-148a, miR-222 and miR-21 may cause fludarabine resistance through inhibiting the activation of p53-responsive genes. Importantly, when the gene expression profile was analyzed, p53 downstream genes were only detected in fludarabine-responsive patients, but not resistant patients [24]. The mutation of TP53 in CLL was associated with unfavorable treatment response and clinical outcome [26], and in some CLL patients inactivation of TP53 correlates with reduced miR-34 expression [27]. In addition to miR-34, 3 other miRNAs-miR-182-5p, miR-7-5p and miR-320c/d-have also been found as p53 targets in CLL [28]. Moreover, miR-132 and miR-212 expression was lower in progressive CLL patients compared with stable CLL patients [29]. Gene expression profiling showed that the miRNAs miR-132 and miR-212 affected the Rb or TP53 signaling pathway, which may explain the clinical observation [29].

The expression of miRNAs can be used as a biomarker to monitor CLL progression. Lower expression of miR-181b, miR-29c and miR-223 was associated with disease progression in CLL patients and this correlates with unfavorable prognosis, such as shorter progression-free survival and overall survival [23, 30–33]. Down-regulation of miR-223 was correlated with the up-regulation of HSP90B1, which was independently predictive of shorter time to the first therapy in CLL patients with unmutated IGHV [33]. The TCL1 oncogene is a target of the miR-29 family and in CLL patients low miR-29c expression was associated with high TCL1 expression [30]. miR-150 was highly expressed in both cellular and serum samples of CLL patients [34]. It is interesting to note, though, that low cellular but high serum expression of miR-150 was associated with poor prognosis as indicated by tumor burden, treatment-free survival and overall survival (OS) [34]. This could be because lower expression of cellular miR-150 may enhance cell survival and proliferation in response to BCR signaling stimulation, which worsens the patient prognosis [34]. Furthermore, high serum miR-150 has been correlated with high lymphocytosis, which contributes to high tumor burden and poor clinical outcome [34].

### Chronic myeloid leukemia (CML)

CML is a hematopoietic malignancy characterized by abnormal expansion of immature hematopoietic progenitor cells in the bone marrow and increased levels of myeloid cells in the peripheral blood. The genetic hallmark of CML is a t (9;22) (q34;q11) reciprocal translocation, also called 'Philadelphia chromosome'. This translocation results in a BCR-ABL fusion gene that leads to constitutive tyrosine kinase activation [35].

#### miRNA signature in CML

The most frequently deregulated miRNAs in chronic myeloid leukemia include miR-10a, miR-17/92, miR-150, miR-203, and miR-328 [36]. miR-17/92 can be both a tumor suppressor and an oncogene depending on the tumor context. The tumor suppressor function of miR-17/92 is mostly explained through targeting pro-survival proteins BCL2, STAT5 and JAK2. On the other hand, targeting of the CDK inhibitor CDKN1A (p21) may explain the oncogenic role of miR-17/92. In a clinical setting, miR-17/92 is up-regulated in early chronic-phase (CP), but not in blast-crisis (BC) CML CD34 (+) cells when compared with normal CD34 (+) cells. In addition, both BCR-ABL and c-Myc up-regulated the expression of miR-17/92 in BCR-ABL-positive cell lines, suggesting it may be used as a therapeutic target [37].

A critical step for the progression to CML blast crisis stage is the down-regulation of miR-328, which is observed in a BCR-ABL dose- and kinase-dependent manner. Ectopic expression of miR-328 in cell lines restored differentiation of leukemic blasts by induction of the survival factor PIM1 and inhibition of the hnRNP E2 interaction with the hematopoietic transcription factor CEBPA. The differentiation arrest observed during the blast phase of CML required the activity of hnRNP E2, a poly (rC)-binding protein, which behaves as a translational regulator [36, 38].

Finally, expression of miR-130a and miR-130b, controlled by BCR-ABL, down-regulated the expression of CCN3, a growth inhibitory protein [39]. Some miRNAs, miR-203 and miR-451, inhibit ABL and BCR-ABL expression directly [40]. Consistent with this notion, miR-203 is frequently silenced by monoallelic loss and hypermethylation of the remaining allele [41]. Another consistency found in CML patients is the reduction of miR-150 and miR-10a expression [42, 43]. CML patients displayed inverse expression levels of miR-150 and the transcriptional activator MYB, which correlates with BCR-ABL (fusion gene) transcript levels [44], while down-regulation of miR-10a was associated with increased proliferation by overexpression of the upstream stimulatory factor 2 (USF2) [42]. More recently, miR-362-5p was found to act as an oncomiR by down-regulating GADD45α, which in turn activated the JNK1/2 and P38 signaling in CML patient samples [45].

#### miRNA expression and drug response in CML patients

As discussed above, the expression of miRNAs can be used as a biomarker to monitor CML progression as well. For example, the expression profile of some miRNAs can predict the Imatinib therapy response in CML patients [46]. Expression of miR-26a, miR-29c, miR-130b and miR-146a was higher in patients with an Imatinib response than in patients with Imatinib-resistant treatment [47]. The potential targets of the miRNAs listed above are c-IAP-1 and MCL1, which are important for tumor cell survival following treatment, while miR-23a, miR-30a, miR-30e, miR-203, miR-320 and miR424 are known to target BCR-ABL [48–52]. Down-regulation of BCR-ABL reduced activation of the pro-survival PI3K/AKT and NF-κB signaling pathway in CML [48]. As a result, high expression of miRNAs targeting BCR-ABL sensitized the CML cells to Imatinib treatment, suppressed proliferation and induced apoptosis [49].

Similarly, loss of miR-217 and miR-199b expression has been correlated with resistance to ABL tyrosine kinase inhibitors [53, 54]. Molecular mechanisms underlying these effects are partly explained by the fact that ectopic expression of miR-217, in tyrosine kinase inhibitor-resistant K562 cells, resulted in reduced expression of DNMT3A and increased the sensitivity of tumor cells to tyrosine kinase inhibitors [54]. It should be noted that the tyrosine kinase inhibitor (TKI) Dasatinib affected miR-let-7d, miR-let-7e, miR-15a, miR-16, miR-21, miR-130a and miR-142-3p expressions, while Imitanib affected miR-15a and miR-130a levels [47]. Consistent with the notion that miR-130a can act as a tumor suppressor by targeting BCL2 and MCL-1 expression, lower expression of miR-130a is associated with poor prognosis as indicated by shorter overall survival and treatment-free survival in CML patients [55]. Importantly, low expression of miR-148b was found in a subset of CML patients with stable complete molecular responses after stopping Imatinib treatment [56]. Using IPA software (Ingenuity System), Ohyashiki found several potential target genes of miR-148b: cholecystokinin B receptor (CCBR), DNA methyltransferase 1 (DNMT1), DNMT3, nuclear receptor subfamily 1, group 1, member 2 (NR112), peroxisome proliferator-activated receptor alpha (PPARA), ribosomal protein S6 kinase, and polypeptide 5 (RPS6KA). Among those target genes, DNMT1 and DNMT3 are known to regulate the expression of FOXO3, which is important for T-regulatory (Treg) cell development. Low expression of miR-148b may cause up-regulation of DNMT and down-regulate the Treg activity, just as Imatinib is known to inhibit Treg activity [56]. These studies suggest that the expression of specific miRNAs can be used to determine a subgroup of CML patients that can safely stop TKI treatments.

### Acute lymphoblastic leukemia (ALL)

ALL is a lymphoid malignancy affecting the B or T lineages. Distinct microRNA signatures are reported in different ALL subtypes and can be used for the diagnosis and classification of ALL. ALL can be divided into T-cell, MLL-rearranged, TEL-AML1-positive, E2A-PBX1-positive, hyperdiploid ALL, BCR-ABL-positive, and “B-other” ALLs. Studies of the distinct microRNA signatures of ALL subtypes can be used for the diagnosis and classification of the disease [57].

#### miRNA signature in ALL

The B and T lineages of ALL can be distinguished based on relative expression of miR-148, miR-151, miR-424, miRNA-425-5p, miRNA-191, miRNA-146b, miRNA-128, miRNA-629, and miRNA-126. In addition, miRNA-708 was found highly expressed in TEL-AML1, BCR-ABL, E2A-PBX1, hyperdiploid, and B-other cases [57, 58]. The miRNA signature in hyperdiploid and TEL-AML1-positive patients partly overlap, suggesting a common underlying biology. Analyses of over 430 miRNAs in 50 clinical T-ALL samples revealed a common signature: miR-223, miR-19b, miR-20a, miR-92, miR-142-3p, miR-150, miR-93, miR-26a, miR-16 and miR-342 [59]. Interestingly, miR-19b,−20a, −26a, −92 and −223 can cooperate with Notch to induce leukemia in a mouse T-ALL model [59]. These five miRNAs have been shown to target T-ALL tumor suppressors such as IKAROS, PTEN, BIM, PHF6, NF1 and FBXW7 [60]. The expression pattern of these miRNAs can be used as a biomarker to distinguish the B and T lineages of ALL. Higher expression of miR-128b and lower expression of miR-223 has independently been reported for human ALL cell lines and ALL cells isolated from pediatric patients [61]. In a different study, single nucleotide polymorphism (SNPs) analyses of precursor miRNAs (pre-miRNA) and miRNA-processing genes revealed eleven SNPs associated with ALL susceptibility [62]. Among them, eight are located at six miRNA biogenesis pathway genes (*TNRC6B*, *DROSHA*, *DGCR8*, *EIF2C1*, *CNOT1*, and *CNOT6*) and three at miRNA genes (*miR-612*, *miR-499*, and *miR-449b*). Interestingly, miRNA-612 and miRNA-499 have significant correlations with ALL susceptibility [57]. In addition, miRNA profiles can be useful to distinguish myeloid or lymphoid lineages of leukemia. De Leeuw et al. identified miRNA-23a, miRNA-27a, miRNA-199b, miRNA-221, and miRNA-223 as the most lineage-discriminative miRNAs between AML and ALL [63, 64]. AML patients present down-regulation of let-7b and miRNA-223 and overexpression of miRNA-128a and -128b compared to ALL. In agreement with these results, Wang et al. [65] identified miR-23a, miR-27a, miR-27b, miR-150, miR-199a, miR-199b, miR-221 and miR-340 as miRNAs differentially expressed in patients with ALL when compared to AML.

#### miRNA expression and drug response in ALL patients

Epigenetic deregulation is one of the mechanisms reported to accelerate ALL disease progression. miR-124a was methylated in 59 % of ALL patients and down-regulation of miR-124a increased the expression of CDK6 resulting in phosphorylation of retinoblastoma (Rb) and increased cell proliferation. As a result, hypermethylation of miR-124a in ALL patientscorrelated with a higher relapse and mortality rate and can be used as an independent prognostic factor for disease-free survival (DFS) and overall survival in the multivariate analysis [66]. microRNA analysis from different studies showed that expression of miR-10a, miR-134, miR-214, miR-221, miR-128b, miR-484, miR-572, miR-580, miR-624 and miR-627 was significantly correlated with a favorable clinical outcome [61, 65, 67]. In contrast, deregulation of the expression of miR-9, miR-33, miR-92a, miR-142-3p, miR-146a, miR-181a/c, miR-210, miR-215, miR-369-5p, miR-335, miR-454, miR-496, miR-518d, and miR-599 was associated with an unfavorable long-term clinical outcome in ALL patients [65, 67–73]. Low expression of miR-151-5p and miR-451, and high expression of miR-1290 or a combination of all three, predicted inferior relapse-free survival (RFS) in pediatric B-ALL [74]. Importantly, activation of NOTCH intracellular domain (NCID) signaling led to transcriptional repression of miR-451 and miR-709, two tumor suppressor microRNAs in T-ALL. In fact, ICN1 decreased expression of these miRNAs by promoting the degradation of the tumor suppressor E2A, which induced the transcription of miR-451 and miR-709. Myc was directly repressed by both miR-451 and miR-709. In addition, miR-709 inhibited the expression of Akt and Ras-GRF1 oncogenes. Consistent with this, repression of miR-451 and miR-709 expression was involved in the initiation and maintenance of mouse T-ALL [75]. Furthermore, different independent analyses identified relapse-related miRNAs. When globally analyzed the relapse-related miRNAs-miR-7, miR-100, miR-216 and let-7i—were up-regulated, and miR-486, miR-191, miR-150, miR-487 and miR-342 were down-regulated in early relapse ALL patients [76]. In addition, overexpression of miR-708, miR-223 and miR-27a has been associated with lower relapse-free survival in patients [77], possibly through regulation of FOXO3, BMI1 and E2F1. Expression of miR-126, miR-345, miR-222, and miR-551a were reduced in ALL patients with central nervous system (CNS) relapse compared to non-CNS-relapsed ALL patients [76]. Furthermore, higher expression of miR-7, miR-198 and miR-633 was found in patients with CNS relapse compared with non-CNS-relapsed ALL [76]. Interestingly, target prediction analysis revealed that some deregulated miRNAs might target neuron function- and neurotransmitter-related genes. For example, Glioma Tumor Suppressor Candidate Region Gene 1 (GLTSCR1), Synaptotagmin (STY1), neuronatin (NNAT) and Synaptic Ras GTPase-activating protein 1 (SYNGAP1) are putative targets of miR-126, miR-222, miR-198 and miR-633, respectively [76]. However, a defined role of these miRNAs in the genesis of CNS leukemia is still unclear.

Glucocorticoids can be used to treat ALL because they induce apoptosis in lymphoid lineage cells [78]. In ALL patients, sensitivity to prednisone treatment is an important indicator for treatment outcome [76]. While miR-16 was lower in ALL patients with low leukocytes and good cytogenetic characteristics [79], higher expression of miR-16 was found in patients with corticosteroid resistance [79] and correlated with shorter disease-free survival and overall survival, possibly by modulating BCL-2 [80]. The expression of miR-223 and the miR-15/16 family was increased in ALL patients treated with systemic glucocorticoid monotherapy [61, 78]. In contrast, the expression of miR-548d-1 and miR-106b ~ 93 was reduced after ALL patients were treated with glucocorticoids [78]. Differential expression of miR-18a, miR-532, miR-218, miR-625, miR-193a, miR-638, miR-550 and miR-633 can be used as a marker to predict prednisone response in pediatric ALL patients [76]. For example, high miR-18a but low miR-193a expression is associated with good prednisone response. Although up-regulation of miR-128a [81, 82] and miR-128b [61] is frequently found in childhood ALL patients, poor prednisolone response is often associated with lower miR-128b expression, while higher expression of miR-128b correlated with a longer disease-free period [61]. Consistent with this finding, miR-128b sensitized MLL-AF4 acute lymphocytic leukemia cells to glucocorticoid treatment [83]. miR-128b is an important glucocorticoid sensitizer in MLL-rearranged ALL and displays a cooperative effect with miR-221. Both miRNAs are commonly down-regulated in MLL-ALL compared with other types of ALL [84]. The chimeric mRNAs, MLL-AF4 and AF4-MLL, which are involved in the initiation of the disease, are important targets of miR-128b. Inhibition of miR-128b expression resulted in reduced steroid sensitivity through increasing expression levels of both oncogenic fusion proteins, however the mechanism used by MLL-AF4 and AF4-MLL to induce resistance to glucocorticoids is not defined. In addition, miR-221 targets CDKN1B, which is transcriptionally activated by both the fusion proteins and the wild type MLL [83]. CDKN1B is an important cell-cycle regulator; induction of CDKN1B by MLL-AF4 and MLL might be involved in the resistance to certain chemotherapy drugs by inducing quiescence [85].

Increased miR-708 expression was detected in childhood ALL with a good response to prednisone and with better remission status after 15-day and 33-day chemotherapy protocol [77]. The expression of let-7e was generally reduced in pediatric ALL patients [81, 82], but higher expression of let-7e has been associated with positive minimal residual disease (MRD) at day 14 after treatment [82]. However, further studies are needed to confirm the relationship between let-7e and ALL clinical outcomes because of the small sample size (*n* = 12).

#### Acute myeloid leukemia

AML presents abnormal miRNA expression diversely expressed in the different subtypes. Both the t (8;21) and inv (16) chromosomal aberration are associated with the formation of novel chimeric fusion genes that involve the core-binding factor (CBF) complex, an important regulator of hematopoiesis, providing the designation CBF-AML [86].

#### miRNA signature in AML

A distinct miRNA signature is characterized by an alteration of miR-29, miR-125, miR-142, miR-146 and miR-155 expression, which has been reported to play a role in AML progression and pathogenesis [87]. miR-29 family members miR-29a, miR-29b, and miR-29c have acted as oncogenes and tumor suppressors in myeloid malignancies [88]. miR-29b targeted DNA methyltransferase DNMT3A, DNMT3B, and Sp1 (a transcriptional regulator of DNMT1) [89]. Inhibition of miR-29b promoted DNA hypermethylation in AML and contributed to methylation status in AML cells, suggesting its potential role as a therapeutic target in AML. In addition, miR-29a and miR-29b affected the expression of genes involved in apoptosis, cell cycle progression, and cellular proliferation. Consistent with this, altered expression of MCL-1 and CDK6 was reported in primary AML blasts following ectopic expression of miR-29b [88]. Interestingly, injection of precursor miR-29b oligonucleotides in mice engrafted with K562 cells reduced their tumor sizes [87].

The miR-125 family includes miR-125a/miR-99b/let-7e, miR-125b-2/miR-99a/let-7c-1, and miR-125b-1/miR-100/let-7a-2 located on human chromosomes 19, 21, and 11, respectively. The miR-125 family is involved in self-renewal, both in hematopoietic stem cells (HSC) and Megakaryocyte-Erythroid Progenitor Cells (MEC) [90]. Overexpression of miR-125 enhances the development of an MPN-like phenotype, which progresses to AML. In acute myeloid leukemia, miR-125b was significantly overexpressed in patient blasts and can promote the transformation of normal hematopoietic cells into malignant cells in an in vitro and in vivo model. In addition, miR-125b targeted tumor suppressor BCL2-antagonist/killer 1 (Bak1) to promote AML cell proliferation and inhibit cell apoptosis [91]. miR-125b is located on chromosome 21 and involved in the development of a rare subtype of AML, acute megakaryocytic leukemia (AMKL), especially in patients with Down’s syndrome (DS). The trisomy chromosome 21, typical of DS, was associated with overexpression of miR-125b in both DS- and non-DS-related AMKL patients [92]. Similarly, down-regulation of miR-146a promoted AML disease progression by TRAF6-mediated induction of NF-κB [93] and miR-142 promoted the development of lymphoid and myeloid leukemia and was found recurrently mutated in AML [94].

miR-155 is located on human chromosome 21 in the B-cell integration cluster (BIC) gene [95], which cooperates with c-Myc to induce lymphomas [95]. In addition, miR-155 inhibited the cell-cycle regulator WEE1 and the mismatch repair genes hMLH1, hMSH2, and hMSH6, resulting in an increase in spontaneous mutation rates in hematopoietic stem and progenitor cells (HSPC) and solid tumor cell lines [87, 96, 97]. Interestingly, the expression level of miR-155 was the same as normal bone marrow in Fms-like tyrosine kinase 3 (FLT3)-wildtype AML and higher miR-155 expression was limited to FLT3-ITD mutation AML [98]. In FLT3-wildtype AML cells, miR-155 induced myelomonocytic differentiation and apoptosis [99] by targeting MEIS1, GF1, c-MYC, JARID2, cJUN, FOS, CTNNB1 and TRIB2. The discrepancy between different subgroups of AML is most likely dependent on the disease context or tissue type.

#### MicroRNAs in the diagnosis of AML

Up-regulated let-7a-2-3p has been associated with a favorable prognosis, longer overall survival and event-free survival in cytogenetically normal AML [100]. High let-7a-2-3p expression was associated with reduced expression of oncogene JDP2 and leukocyte immunoglobulin-like receptor (LILRA5/6, LILRB2/3), which are correlated with poor prognosis in cytogenetically normal acute myeloid leukemia (CN-AML) patients [100]. In addition, up-regulated let-7a-2-3p was correlated with down-regulation of the ERBB signaling pathway and JAK-STAT signaling pathway. High miR-181 expression is also associated with a better clinical outcome in CN-AML [101, 102] through reverse regulation of toll-like receptors and interleukin-1β. In addition, miR-181 contributed to a better clinical outcome in cytogenetically abnormal AML patients [103] by regulation of HOXA7, HOXA9, HOXA11, and PBX3. Drug resistance is the main reason for AML relapse and poor prognosis and, since miR-181b can increase AML drug sensitivity through down-regulation of HMGB1 and MCL-1, it is not surprising that miR-181b was down-regulated in relapsed and refractory AML patients [104].

#### MicroRNA expression associated with favorable prognosis in AML

In analysis of the expression of the meningioma 1 (MN1) gene and MN1-associated microRNA in Chinese adult de novo acute myeloid leukemia (AML) patients, Xiang found that increased expression of MN1 was associated with reduced miR-20a expression and increased miR-181b expression. In addition, miR-20a up-regulation was also associated with a higher complete remission rate and longer overall survival [105]. In contrast, high miR-181b expression was found in patients with a lower complete remission rate, shorter relapse-free survival and shorter overall survival [105].

Cytogenetic risk factors and molecular markers are important factors for AML prognosis [106]. Expression signatures of a minimum of two (miR-126/126*), three (miR-224, miR-368, and miR-382), and seven (miR-17-5p and miR-20a, along with the aforementioned five) miRNAs could correctly distinguish CBF, t (15;17), and MLL-rearrangement AMLs, suggesting that these microRNAs may cooperate with specific translocation in leukemogenesis [107]. In fact, KIT-mediated up-regulation of miR-17, which targets RUNX1-3'UTR, mimicked the effects of the CBF-AML fusion protein [108]. The expression of miR-29a was lower in the bone marrow of pediatric AML patients compared with normal controls [109], and reduced miR-29a expression was associated with unfavorable karyotypes and shorter relapse-free and overall survival in pediatric AML patients [109]. Importantly, the association of miR-29a and prognosis was more apparent in intermediate-risk cytogenetic AML patients [109]. The same is true for miR-29b in that AML patients with low miR-29b expression had an unfavorable overall survival [110].

Analyses of 238 intermediate-risk cytogenetic AML patients showed that reduced expression of miR-135a and miR-409-3p is associated with a higher risk of relapse [106], while higher miR-142 expression was associated with a better overall survival in these same patients [111]. miR-142 was highly expressed in mature hematopoietic cells and had an essential role during T-lymphocyte development although its role in leukemogenesis is unclear.

Another miRNA associated with a favorable prognosis was miR-34a, a microRNA regulated in a p53-dependent and -independent manner. In AML patients with complex karyotype, p53 status played a role in determining miR-34a’s role in clinical prognosis. Up-regulation of miR-34a expression was correlated with unfavorable overall survival in TP53 (unaltered)-AML with complex karyotype, but was correlated with favorable overall survival and chemotherapy sensitivity in TP53 (biallelic altered)-AML with complex karyotype [112]. In analyzing p53 pathway genes, Rücker didn’t find a correlation between p53 pathway genes and miR-34a expression, which indicated p53-independent miR-34a induction [112].

Furthermore, miR-96 is down-regulated in newly diagnosed AML patients and is associated with a higher white blood cell (WBC) count, bone marrow blast count, and lower hemoglobin and platelet count. Importantly, the expression of miR-96 increased after patients were treated with standard cytarabine plus daunorubicin induction chemotherapy [113]. When analyzing the relapse-free survival and overall survival, low expression of miR-96 was associated with shorter RFS and OS [113].

miR-204 expression was reduced in AML patients and low miR-204 expression was correlated with short patient survival [114]. After patients received induction chemotherapy (daunorubicin plus cytarabine), high expression of miR-204 was associated with complete remission [114]. miR-204 targeted HOXA10 and MEIS1 genes [115], which perturb myeloid differentiation and might lead to AML. In addition, increased expression of miR-212, miR-25 and/or miR-203 has been associated with a favorable overall survival, event-free and relapse-free survival in AML patients independent of cytogenetic subtypes [65, 116–118]. The reason why these miRNAs are associated with a favorable prognosis is unclear, although miR-25 is reported to be involved in cell migration and dissemination by targeting αv- and α6-integrin. Ectopic expression of miR-25 resulted in inhibition of migration in high motility cells [119]. Interestingly, AML patients with high expression levels of miR-212 displayed a significant enrichment of genes involved in the immune response. Consistently, CCL3 and CCL4 were found up-regulated in high miR-212 expression cases and, as genes that belong to the CCL2-4/CXCL1/8 class of chemokines, they are involved in T- and NK-cell chemotaxis. An enhanced chemotaxis of immune cells might contribute to their anti-leukemic effects and result in a better response to chemotherapy treatment in patients with high miR-212 expression [65, 116–118]. Finally, miR-223 was found to inhibit cellular growth in leukemia cells by targeting Insulin-Like Growth Factor 2 (IGF2) [120].

#### miRNA expression associated with unfavorable prognosis in AML

Different studies identify miRNAs deregulated in AML, which are associated with an unfavorable prognosis. miR-378 was increased in 31 % of AML patients and was associated with lower hemoglobin levels and shorter relapse-free survival [121]. There was a positive correlation between miR-155 expression and white blood cell (WBC) count, serum lactate dehydrogenase (LDH), C-reaction protein (CRP) value in peripheral blood (PB), and miR-25 and miR-196b expression in AML [122]. miR-126 was highly expressed in hematopoietic stem cells and leukemic stem-like cells and in AML patients high miR-126 expression was correlated with poor survival, higher chance of relapse and induced higher expression of stem cell-related genes [123, 124]. In vitro, overexpression of miR-126-5p increased the phosphorylation of Akt and caused cytarabin resistance. Increased miR-124, miR-128-1, miR-194, miR-219–5p, miR-220a and miR-320 expression are associated with increased risk in AML, however the role of microRNAs in the development of AML is unclear [101]. The expression of miR-320d was increased in AML patients [125] and higher expression of miR-124-1 was associated with shorter overall survival and relapse-free survival [126]. Along these lines, AML patients with worse overall and event-free survival were known to have higher expression of miR-191 and miR-199a [127].

In de novo AML patients, miR-9-5p and miR-155-5p were independent unfavorable prognostic factors [117]. miR-155 was up-regulated in AML patients compared to normal controls [122, 125] and this high expression was associated with the aforementioned unfavorable prognosis, including lower complete remission rate and shorter disease-free survival and overall survival in AML [117, 122, 128]. The deregulation of miR-155 was associated with a gene expression profile enriched for genes involved in apoptosis, nuclear factor-kappaB activation, and inflammation [128].

Analyzing 53 AML patients, increased expression of miR-26a, miR-29b, miR-146a, and miR-196b was associated with an unfavorable overall survival [65]. The role of miR-196b was further supported by analyzing 238 intermediate-risk cytogenetic AML patients, whereby high miR-196b and miR-644 expression was associated with shorter overall survival [106]. In 40 non-M3 AML patients, high expression of miR-26a, miR-29b and miR-146a was associated with short overall survival [65]. It is worth noting that miR-146a expression was reversely correlated with prognosis in both ALL and AML patients [65] while the opposite role of miR-29b in AML prognosis has been reported. miR-29b expression was inversely associated with MLLT11 expression, which is a poor prognostic biomarker for AML patients. Low miR-29b and elevated MLLT11 expression are found in patients with poor overall survival [110], but whether the cooperation between miR-29b and MLLT11 caused the poor prognosis needs to be further confirmed.

Reduced miR-188-5p expression was associated with a favorable prognosis as indicated by longer overall survival and event-free survival in cytogenetically normal AML patients [100]. Low miR-188-5p expression was associated with up-regulation of FOSB, small nucleolar family (SNORD50A, SNORD105, SNORD11B) and zinc finger protein in AML patients. FOSB is known to regulate cell proliferation and differentiation by acting as a cofactor for the JUN family. Up-regulated miR-3151 was found in AML patients with an unfavorable prognosis, such as short overall survival and leukemia-free survival, and higher relapse risk [129, 130]. High expression of miR-3151 was associated with a low expression of genes involved in transcriptional regulation, posttranslational modification, and cancer pathways, such as FBXL20 and USP40 [130]. High miR-3151 expression was associated with high miR-501-5p and low miR-590, miR-135a, miR-100*, miR-186* and let-7a* expression, however the significance of this association is unknown [129]. The expression of let-7a-3 was increased in 25 % of de novo AML patients and was associated with shorter overall survival and relapse-free survival [131] in AML patients with complete remission. Further studies are needed to confirm the role of let-7a-3 and let-7a-2-3p in AML.

#### miRNA expression and drug response in AML patients

Higher expression of miR-29b was found in older AML patients with single-agent decitabine response compared with non-response patients [132]. The ability of miR-29b to target DNA methyltransferases may explain the role of miR-29b in decitabine response. miR-29c expression was higher in AML patients compared with healthy controls and was associated with poor survival [133]. Reduced miR-29c expression was associated with complete remission after initial treatment (intensive chemotherapy: daunorubicin plus cytarabine or low dose chemotherapy (low dose cytarabine or azacitidine)). Higher miR-29c expression was associated with relapse after patients achieved complete remission. Importantly, low miR-29c expression was associated with better response to azacitidine treatment and remission achievement in elder AML patients who were not suitable for intensive chemotherapy [133].

The increased expression of miR-181a was associated with a higher complete remission rate, longer overall survival and disease-free survival [102, 103] in AML patients treated similarly with intensive induction chemotherapy and consolidation with autologous peripheral blood stem-cell transplantation on Cancer and Leukemia Group B (CALGB) protocols 9621 and 19,808. Recent evidence has shown that miR-181a is a negative regulator of NF-κB signaling in diffuse large B-cell lymphoma (DLBCL). Overexpression of miR-181a decreased tumor cell proliferation and survival, suggesting its role as a prognostic marker [134]. In addition, after AML patients received double induction and one consolidation therapy, increased miR-181b expression was associated with worse complete remission rates, relapse-free survival and overall survival in adult patients with de novo AML [105]. In vivo study showed the capability of miR-181b to reduce leukemic cell expansion and to increase survival of treated mice. miR-181b affected the expression of TCL1, Bcl2 and Mcl1 anti-apoptotic factors, and reduced the levels of Akt and phospho-Erk1/2 [135].

While up-regulation of miR-20 was associated with higher complete remission rates and overall survival [105], miR-204 expression was reduced in AML patients and low miR-204 expression was correlated with short patient survival [114]. After patients received induction chemotherapy (daunorubicin plus cytarabine), high expression of miR-204 was associated with complete remission [114]. A possible explanation is that mir-204 inhibits cell proliferation by targeting the transcription factor SOX4, which was reported in T-cell acute lymphoblastic leukemia [136].

miR-331 is up-regulated in AML patients and AML patients with longer complete remission after induction chemotherapy had reduced miR-331 expression [137]. In agreement with this notion, miR-331 promoted proliferation and metastasis in other cancer types by targeting PHLPP, resulting in stimulation of protein kinase B (AKT) [138]. miR-335 was up-regulated in pediatric AML patients both in bone marrow and serum [139], and high serum miR-335 was associated with poor relapse-free and overall survival after patients received 10 days of induction chemotherapy [139]. In addition, high expression of miR-335 in the bone marrow was indicative of poor Ara-C-based chemotherapy response, lower relapse-free survival and overall survival in AML patients [140]. Interestingly, miR-335 was reported to be involved in the regulation of target genes in several oncogenic signaling pathways, such as p53, MAPK, TGF-b, Wnt, ERbB, mTOR, Toll-like receptor and focal adhesion [141]. High expression of the miR-10 family was associated with complete remission after AML patients received induction chemotherapy [142, 143]. Finally, the role of miR-10 is still unclear in an AML context.

### Adult T-cell Leukemia (ATL): signature and prognosis

ATL is a fatal malignancy of mature CD4+, CD25+ T lymphocytes induced by the retrovirus Human T-cell leukemia virus (HTLV)-1 [144, 145]. Several studies have reported deregulated microRNAs in ATL patient samples and HTLV-1-transformed cells, among them miR-155, miR-146a, miR-150, and miR-223 were found up-regulated and miR-31 and miR124a down-regulated [146–149].

The expression of miR-223 is known to affect T lymphopoiesis and granulocytosis with up to 20 % of acute ATL patients being diagnosed with the latter, which may be linked to high miR-223 expression [146]. Similar effects of miR-150 have been reported. Ectopic expression of miR-150 in hematopoietic stem cell progenitors reduced mature B cells and enhanced T lymphopoiesis. In HTLV-1-transformed cells, Tax can up-regulate the expression of miR-146a via the NF-κB dependent signaling pathway and the potential targets for miR-146a are IRAK6 and TRAF, which are involved in immune response [147]. miR-31 was down-regulated in ATL via epigenetic regulation and it caused up-regulation of its target gene, NIK, which activated the NF-κB signaling pathway and caused apoptosis resistance [148]. Interestingly, a recent study demonstrated that the virus-encoded protein HBZ targets the expression of DICER, thereby modulating the expression of a subset of microRNAs [150]. Deregulation of miR-146a, miR-155, miR-150 and miR-223 was reported to affect cellular proliferation [151–153] and alteration of miR-31, miR-130b and miR-93 were involved in apoptosis resistance [154], suggesting a possible role of miRNA expression in ATL progression and pathogenesis. Differential analyses of microRNA expression in non-infected healthy individuals, chronic ATL patients and acute ATL patients revealed an increased number of up-regulated miRNAs in acute ATL patients when compared with chronic ATL patients [155]. Among these, increased miR-155 expression correlated with disease progression from HTLV-1 carrier to chronic ATL and then to acute ATL [155]. Both STAT3 and Myb, which transcriptionally up-regulate miR-155, were activated in HTLV-I-transformed cells and ATL samples [149, 156, 157]. miR-155 plays a role in dendritic and T-cell interaction, which is important for early stage infection. In addition, miR-155 also promotes T-helper type 1 (Th1) versus type 2 (Th2) differentiation, which can explain the susceptibility to parasite infection, such as Strongyloides in ATL patients [146]. On the other hand, let-7 g was highest in healthy donors and its expression was significantly reduced in an HTLV-1 carrier, and chronic and acute ATL patient samples [155]. For clinical outcomes, high miR-155 and low miR-126 was associated with a poor prognosis [155]. High miR-155 can reduce TGFβR2 function and increase tumor growth. On the other hand, low miR-126 increased the expression of EGFL7, Crk or SLC7A5, which promote tumor growth. High miR-130b and low miR-145 and miR-223 expression in aggressive-type ATL were associated with shorter overall survival. Among miR-130b, miR-145 and miR-223, only miR-145 can act as an independent risk factor for ATL prognosis by a multivariate prognostic analysis. An in vitro study showed that overexpression of miR-145 in ATL cells reduced cell proliferation [158]. A recent study demonstrated that epigenetic silencing of miR-124-1 resulted in STAT3-mediated Pim1 kinase activation and increased tumorigenic potential [149].

### Role of circulating RNA

The majority of miRNAs are cellular miRNAs, however an emerging class of circulating miRNAs has been described. Circulating miRNAs have been observed in various body fluids [159] and are involved in cellular proliferation, differentiation and disease progression or diagnosis (Table 3). There are several advantages to analyzing circulating RNA instead of malignant cellular RNA. First, body fluids like urine and saliva are easier to collect than malignant cells. Furthermore, exosomes in urine are stable for up to a week at room temperature [160]. Second, in addition to malignant cells, circulating RNA can affect the microenvironment and other distant organisms.

**Table 3:** Circulating microRNAs in human leukemia

Circulating miRNA	Expression in patients	Association with blood cell count	Predict prognosis	Predict Disease progression	Predict Drug response
miR-29	up in CLL exosome	Yes	Yes	–	–
miR-150	up in CLL exosome, low in AML plasma	Yes	Yes	–	Yes
miR-155	up in CLL exosome, up in AML plasma	Yes	Yes	Yes	Yes
miR-181b-5p	up in AML plasma	–	Yes	–	Yes
miR-210	up in AML serum	–	Yes	–	Yes
miR-375	up in AML serum	–	Yes	–	–
miR-511	up in B-ALL plasma	–	–	–	–
miR-222	up in B-ALL plasma	–	–	–	–
miR-34a	up in B-ALL plasma	–	–	–	–
miR-199a-3p	low in B-ALL plasma	–	–	–	–
miR-223	low in B-ALL plasma	–	–	–	–
miR-221	low in B-ALL plasma	–	–	–	–
miR-26a	low in B-ALL plasma	–	–	–	–
let-7d	low in AML plasma	–	–	–	–
miR-339	low in AML plasma	–	–	–	–
miR-342	low in AML plasma	–	Yes	–	–
let-7b	up in AML plasma	–	–	–	–
miR-523	up in AML plasma	–	–	–	–
miR-328	low in AML plasma	Yes	Yes	–	–

The table reports the circulated microRNAs that have been identified in human leukemia (CLL, CML, ALL, AML and ATL) and their role in prognosis, disease progression, drug response or association with blood cell count

Circulating miRNA and cellular miRNA expression profiles may also differ. For instance, analyses of miRNA in CLL plasma suggested the presence of miR-155, but intracellular miR-155 was not detected. Plasma miR-155 can be used to predict overall survival in CLL patients and this difference may come from the plasma pool of miRNAs having various cellular sources [15]. Finally, deregulation of miRNA expression can happen at early stages of tumorigenesis and measuring circulating miRNA levels can be useful for early cancer detection and improving patient survival [161]. In addition, malignant cells are usually reduced after treatment, whereas the circulating RNAs can still be detected.

Recent evidence showed elevated expression of the miR-29 family (miR-29a, miR-29b and miR-29c), miR-150 and miR-155 in CLL-derived exosomes compared to healthy donors [162]. The plasma expression of miR-29a and miR-150 was associated with absolute lymphocyte count in the blood [163]. The miR-29 family was significantly down-regulated in a subset of CLL patients and was associated with an unfavorable prognosis [7]. miR-150 was highly expressed in cellular and serum samples of CLL patients [34] and, interestingly, low cellular expression of miR-150 but high serum expression of the same was associated with poor prognosis as indicated by tumor burden, treatment-free survival and overall survival [34]. The expression of miR-155 was increased with disease progression from monoclonal B-cell lymphocytosis (MBL) to CLL and was higher in MBL and CLL than normal controls [15]. In addition, high plasma miR-155 expression was associated with CLL patients poorly responding to fludarabine, cyclophosphamide, and rituximab (FCR) chemotherapy [15]. Therefore, high expression of miR-155 was associated with more aggressive disease and poorer clinical prognosis in CLL [16, 17].

There was a positive correlation between miR-155 expression and white blood cell count, serum lactate dehydrogenase (LDH) and C-reaction protein (CRP) value in peripheral blood in AML patients [122]. High miR-155 expression was associated with an unfavorable prognosis, such as lower complete remission rate and shorter disease-free survival and overall survival in AML patients [117, 122, 128]. Using TaqMan miRNA microarray and quantitative real-time RT-PCR, Fayyad-Kazan found that the expression of let-7d, miR-150, miR-339, and miR-342 was down-regulated, and let-7b, and miR-523 was up-regulated in AML patient plasma compared to normal controls. Up-regulation of miR-150 and miR-342 after treatment was associated with AML patients with complete remission [164]. In addition, circulating miR-155-5p and miR-181b-5p were up-regulated in AML patients when compared with normal controls [125]. Up-regulated circulating miR-181b-5p was associated with shorter overall survival [125] and was found in patients with a lower complete remission rate, shorter relapse-free survival and shorter overall survival [105]. Other circulating miRNAs can also act as biomarkers for AML prognosis. For instance, miR-210 was up-regulated in the bone marrow and serum of AML patients compared with normal controls. Reduced serum miR-210 expression was found in patients with complete remission, while high miR-210 expression was correlated with poor relapse-free survival and overall survival in AML patients [165]. Similarly, the expression of miR-375 was higher in the serum and bone marrow of pediatric AML patients and was associated with unfavorable karyotypes and poor prognosis as indicated by shorter relapse-free survival and overall survival [166]. Like miR-29a [109], the association of miR-375 and prognosis was more apparent in intermediate-risk cytogenetic AML patients [166]. Plasma miR-511, miR-222, and miR-34a were up-regulated in B-ALL patients compared with normal controls, whereas plasma miR-199a-3p, miR-223, miR-221, and miR-26a were lower in B-ALL patients [167]. Together these studies clearly demonstrate that detection of circulating miRNAs has significant value for detection of disease progression and can also serve as an indicator of therapeutic response.

### Clinical significance of other non-coding RNAs: lncRNA and snoRNAs

In addition to microRNA, other non-coding RNAs have been reported to play a role in human leukemias. Long non-coding RNAs (lncRNAs) are RNA molecules longer than 200 nucleotides with undefined open reading frames involved in gene expression regulation. A small subset of lncRNAs have been reported in leukemia and an lncRNA expression profile correlated with treatment response and survival in AML patients [168]. The X-inactive specific transcript Xist lncRNA, involved in epigenetic regulation of transcriptionally inactive chromatin, was overexpressed in some leukemias [169]. NOTCH-regulated lncRNA LUNAR1 (leukemia-induced non-coding activator RNA) has been shown to have oncogenic effects in T-ALL and has been demonstrated to increase IGF1R mRNA expression and IGF1 signaling [170]. Another NOTCH-related lncRNA, RP11-611D20.2 (NOTCH-associated lncRNA in T-ALL (NALT)), has been found to be overexpressed in pediatric ALL and may play a role in the leukemia stem cell compartment [171]. In CML patients with BCR-ABL translocation, deregulation of two lncRNAs has been described: the Beta Globin Locus 3 (BGL3) lncRNA [172] and the imprinted H19 lncRNA [173]. Little is known about the function of these lncRNAs in CML. BGL3 lncRNA has been shown to increase the expression levels of the tumor suppressor PTEN by acting as a competing endogenous RNA (ceRNA) [174]. In contrast, lncRNA H19, which is transcriptionally activated by the oncogene c-Myc [173], has been shown to inhibit the expression of the onco-suppressor let-7 microRNA family [175]. In AML patients, lncRNA IRAIN [176], which is transcribed from the IGF1R imprinted locus, is down-regulated in patients with high-risk AML, while urothelial carcinoma-associated 1 (UCA1) lncRNA is specifically up-regulated in AML [177], although its role in the pathogenesis is unclear. Finally, the lncRNA B-ALL-associated long RNAs-2 (BARL-2) was found to affect B-ALL patient response to corticosteroid treatment [178]. By using small RNA sequencing, Blume found that long non-coding RNAs (lncRNAs) nuclear enriched abundant transcript 1 (NEAT1) and long intergenic non-coding RNA p21 (lincRNA-p21) are p53 targets in CLL when cells respond to DNA damage. The induction of NEAT1 and lincRNA-p21 were important for p53-dependent cell death after DNA damage [28].

Another class of non-coding ncRNAs, the small nucleolar snoRNAs, is also affected in cancers and leukemia. Elevated levels of SNORD112–114 snoRNAs have been found in acute promyelocytic leukemia (APL) [179]. In a different study, Affymetrix GeneArray screening identified snoRNA SNORA70F as significantly down-regulated in poor prognostic subgroups of CLL patients. In addition, high expression of SNORA74A and SNORD116-18, and low expression of SNORD56, were associated with shorter progression-free survival (PFS) in these patients [180]. Although lncRNA and snoRNA are not as greatly studied as miRNA, they are likely to play an increasing role in the future and eventually become a part of patients’ genetic signatures for individualized targeted medicine.

## Conclusions

Cellular and circulating miRNAs are aberrant in various human cancers [181–184] and can be used as markers for disease diagnosis, progression, treatment response and clinical outcome. The advance in Next-Generation Sequencing has provided us more details on how miRNA and lncRNA deregulation lead to leukemia onset and progression. Since each miRNA or lncRNA regulates multiple genes and signaling pathways, it is believed that the effects of miRNAs and lncRNAs are the combinational output. Therefore, it is important to determine the critical signaling pathway leading to leukemia and identify potential therapeutic treatments. However, there is a silver lining. For example, miR-34 is known to target more than 24 different oncogenes involved in cell proliferation, drug resistance and metastasis. In 2013, Texas–based Mirna Therapeutics launched it phase clinical trial for a miR-34 mimic: MRX34 [185]. The broad effect of MRX34 may prevent drug resistance, which is commonly observed in clinical treatment, and restore cell signaling pathways back to normal. For miRNAs and lncRNAs upregulated in leukemia, small interfering RNAs and antisense oligonucleotides are showing promising results in targeting lymphoma and solid tumors. Although the delivery of a miRNA mimic, small interfering RNAs and antisense oligonucleotides to patients is still challenging, future technical improvements will provide more opportunities for treatment.
